# IMPACT OF ALTERED ROUTINES ON SOCIAL AND MENTAL HEALTH AMONG OLDER ADULTS DURING THE COVID-19 PANDEMIC

**DOI:** 10.1093/geroni/igae098.2950

**Published:** 2024-12-31

**Authors:** Jessica Finlay, Lindsay Kobayashi, Rebecca Milan, Mallory Sagehorn, Grace Bowman, Rohini Perera

**Affiliations:** University of Colorado Boulder, Boulder, Colorado, United States; University of Michigan, Ann Arbor, Michigan, United States; University of Michigan, Ann Arbor, Michigan, United States; University of Colorado Boulder, Boulder, Colorado, United States; University of Colorado BioFrontiers Institute, Boulder, Colorado, United States; University of Michigan, Ann Arbor, Michigan, United States

## Abstract

The COVID-19 pandemic has altered the daily lives of older adults, necessitating adaptations in social, creative, and exercise routines while highlighting the importance of shared ‘third places’ (sites outside of home and work/school) as vital community support. This mixed methods study delves into the nuanced relationship between lifestyle shifts and mental health outcomes among older Americans. The COVID-19 Coping Study’s national three-year follow-up survey was conducted April-May 2023 among 1,915 participants aged 59 years and above. The Beck Anxiety Inventory, Center for Epidemiologic Studies Depression Scale (CES-D), and UCLA Loneliness Scale assessed anxiety, depression, and loneliness. Reflexive thematic analysis of open-ended participant responses investigated underlying motivations and experiences driving lifestyle changes. Participants, predominantly Non-Hispanic White (93%), female (71%), and college-educated (86%), reported significant modifications to their routines influenced by viral exposure concerns, personal health considerations, and broader societal challenges since the pandemic onset. Changes in social routines were most prevalent (52%) and significantly correlated with increased experiences of anxiety (β=0.30, p=0.02), depression (β=0.33, p< 0.01), and loneliness (β=0.30, p< 0.01). Once vital hubs for social interaction and civic engagement, our qualitative findings identified that fear of infection and societal tensions led many to withdraw from shared community and civic ‘third places’. As older Americans navigate the ongoing challenges of COVID-19 and other infectious diseases, understanding the complex interplay between altered lifestyle routines and community support is paramount. Results may inform interventions to develop more inclusive and accessible interventions that foster resilience and wellbeing in later life.

